# Camera-Based Net Avoidance Controls of Underwater Robots

**DOI:** 10.3390/s24020674

**Published:** 2024-01-21

**Authors:** Jonghoek Kim

**Affiliations:** System Engineering Department, Sejong University, Seoul 5006, Republic of Korea; jonghoek@gmail.com

**Keywords:** underwater robot, net avoidance controls, camera-based net detection, reactive control laws

## Abstract

Fishing nets are dangerous obstacles for an underwater robot whose aim is to reach a goal in unknown underwater environments. This paper proposes how to make the robot reach its goal, while avoiding fishing nets that are detected using the robot’s camera sensors. For the detection of underwater nets based on camera measurements of the robot, we can use deep neural networks. Passive camera sensors do not provide the distance information between the robot and a net. Camera sensors only provide the bearing angle of a net, with respect to the robot’s camera pose. There may be trailing wires that extend from a net, and the wires can entangle the robot before the robot detects the net. Moreover, light, viewpoint, and sea floor condition can decrease the net detection probability in practice. Therefore, whenever a net is detected by the robot’s camera, we make the robot avoid the detected net by moving away from the net abruptly. For moving away from the net, the robot uses the bounding box for the detected net in the camera image. After the robot moves backward for a certain distance, the robot makes a large circular turn to approach the goal, while avoiding the net. A large circular turn is used, since moving close to a net is too dangerous for the robot. As far as we know, our paper is unique in addressing reactive control laws for approaching the goal, while avoiding fishing nets detected using camera sensors. The effectiveness of the proposed net avoidance controls is verified using simulations.

## 1. Introduction

Nowadays, underwater robots are applied in various scenarios, such as underwater exploration with or without human intervention [1,2,3,4,5,6,7,8]. The efficiency of underwater robots can improve significantly, and we can reduce the human intervention needed on robot controls.

This paper considers the case where an underwater robot needs to reach its goal without human intervention. In this case, the robot may become tangled by underwater nets and be disabled. Thus, avoiding underwater nets is critical for the reliable maneuvers of an underwater robot. This paper proposes how to make the robot reach its goal, while avoiding fishing nets that are detected using the robot’s camera sensors.

This article considers the case where an underwater robot uses passive cameras for sensing its surrounding environments [9,10,11,12,13]. We argue that passive sensing is more desirable than active scanning methods (e.g., active sonar sensors [14]), since passive sensing consumes much less energy compared to emitting sonar pings. Moreover, if we consider a military underwater robot whose mission is to explore an enemy territory, then it is desirable to operate the robot in a stealthy manner. Therefore, passive camera sensing is more desirable than emitting signal pings continuously. Thus, in our paper, the robot uses passive cameras for the detection of underwater nets.

We can use deep neural networks for the detection of underwater nets based on camera sensor measurements. As deep neural networks, one can use the R-CNN family, which includes Fast R-CNN [15], Faster R-CNN [16], and Mask R-CNN [17], which have both object detection and instance segmentation capabilities. As state-of-the-art deep neural networks, one can use You Only Look Once (YOLO) algorithms [18,19,20], which have been widely used for object detection and bounding box generation.

There are many papers on underwater object detection and inspection using camera sensors [10,11,12,13]. Underwater object detection is not trivial, since light propagation in underwater environments suffers from phenomena such as turbidity, absorption and scattering, which strongly affect visual perception [21]. The reference [11] used color, intensity, and light transmission information for underwater object segmentation. The reference [12] reviewed many papers on underwater object detection methods that have been developed thus far. The reference [13] presented an algorithmic pipeline for underwater object detection and, in particular, a multi-feature object detection algorithm to find human-made underwater objects. In practice, an underwater camera can detect various objects, since there are many living creatures in underwater environments. Instead of detecting and classifying all underwater objects, our approach is to detect underwater nets based on camera sensors. This is due to the fact that avoiding underwater nets is critical for safe maneuvers of an underwater robot.

As far as we know, our paper is unique in proposing how to make the robot reach its goal while avoiding fishing nets detected using camera sensors. Using the camera measurements, we can apply deep neural networks, such as YOLOv5 [19], which compute an image bounding box containing the net. Based on the net’s bounding box, we develop novel reactive control laws for avoiding collision with the detected net.

**Figure 1:**
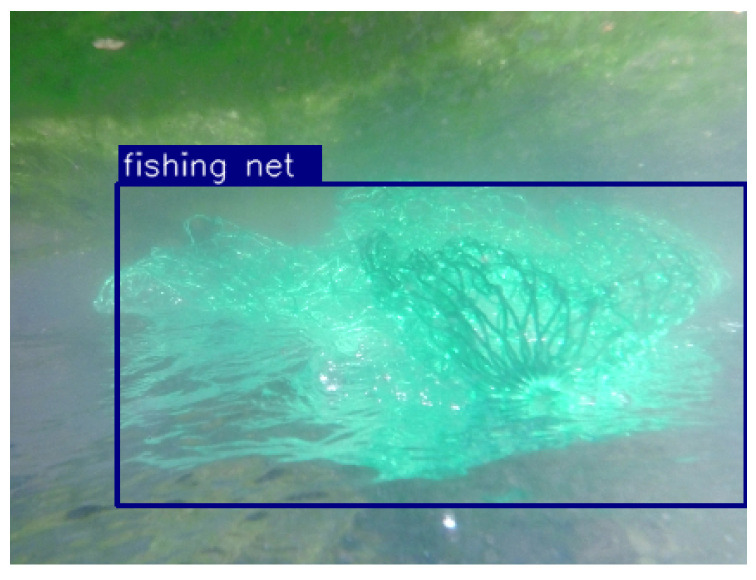
For example, this plot shows the net detection results of YOLOv5. See that a bounding box is generated on the image of an underwater net. The center of a bounding box indicates the bearing angle of a net, with respect to the robot’s camera.

For example, Figure 1 shows the detection results of YOLOv5, in order to detect an underwater net in a camera image. In order to generate datasets of underwater nets, we perform experiments in the stream. The center of a bounding box indicates the bearing angle of a net, with respect to the robot’s camera. Camera images cannot generate the relative distance information between the robot and a net. Camera sensors only yield the bearing angle of a net, with respect to the robot’s camera.

There are many papers on developing collision evasion controls [22,23,24,25]. In  [26], an obstacle avoidance study of a wave glider in a 2D marine environment was conducted. Considering 2D environments, Artificial Potential Field (APF) has been widely used for avoid collision between a robot and obstacles  [26,27]. APF is generated by the addition of the attraction potential field (generated by the goal) and the repulsive potential field (generated by obstacles). Velocity Obstacle (VO) methods can be utilized for collision evasion with moving obstacles [22,24,28,29,30,31]. References [31,32,33] considered collision avoidance in three-dimensional environments.

The collision avoidance methods in the previous paragraph provide the safe motion of a robot, in the case where an obstacle is detected by the robot’s sensor. Moreover, these collision avoidance methods assume that the distance between the robot and an obstacle can be accurately measured. To the best of our knowledge, collision avoidance methods in the literature assumed that the distance between the robot and an obstacle can be accurately measured. This implies that the collision avoidance controls in the literature require that the robot has range sensors.

Our paper considers passive camera sensors that do not provide the distance information between the robot and a detected net. Camera sensors only measure the bearing angle of a net, with respect to the robot’s camera pose. As far as we know, our paper is novel in developing reactive control laws for avoiding collisions based on the net’s bearing angle, which is computed based on the net’s bounding box.

In practice, the detection of underwater nets using camera images is not trivial. For instance, there may be trailing wires that extend from a net, and the wires can entangle the robot before the robot detects the net. Moreover, light, viewpoint and sea floor condition can decrease the net detection probability in practice. Light propagation in underwater environments suffers from turbidity, absorption and scattering, which strongly affect visual perception [21]. Thus, whenever a fishing net is detected using camera image, it is desirable to move away from the detected net abruptly.

In the proposed net avoidance controls, the robot moves away from a detected net whenever it detects a net. Here, the image bounding box position of the net is used to derive the bearing angle of the net with respect to the robot’s camera. Then, the bearing angle is used to make the robot move away from the detected net.

**Figure 2:**
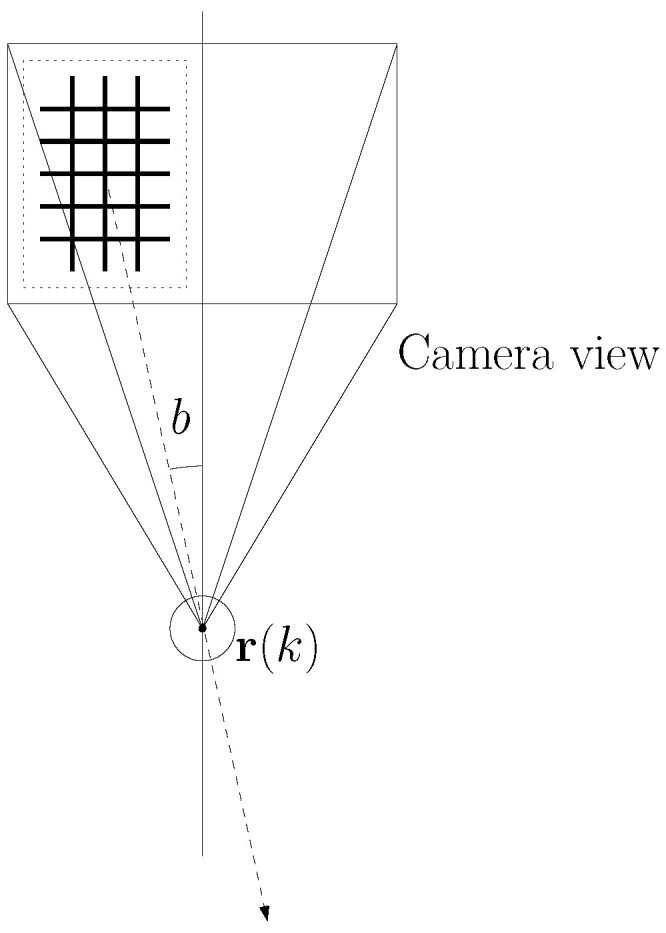
An example of the bearing angle of the net with respect to the robot’s camera. Here, *b* denotes the bearing angle of the net with respect to the robot’s camera. The bold plaid indicates the net in the camera image. A bounding box containing the detected net is plotted with a dotted rectangle. In order to move away from the detected net, the spherical robot at r(k) moves in the dashed arrow direction.

See Figure 2 for an example of the bearing angle of the net with respect to the robot’s camera. In this figure, *b* denotes the bearing angle of the net with respect to the robot’s camera. The bold plaid indicates the net in the camera image. A bounding box containing the detected net is plotted with a dotted rectangle. In order to move away from the detected net, the spherical robot at r(k) moves in the dashed arrow direction.

After the robot moves backward for a certain distance, the robot makes a large circular turn to approach the goal, while avoiding the net. A large circular turn is used, since moving close to a net is dangerous for the robot. The proposed net avoidance controls are simple, and are suitable for real-time embedded system applications.

To the best of our knowledge, our article is novel in addressing reactive control laws for approaching the goal, while avoiding nets detected using passive cameras. The effectiveness of our net avoidance controls is verified using computer simulations.

This paper is organized as follows. Section 2 presents the net avoidance controls, so that the robot can reach its goal without being entangled by underwater nets. Section 3 presents the simulations of our paper. Section 4 provides the conclusions.

## 2. Net Avoidance Controls

Before presenting our net avoidance controls, we address the motion model of the robot. This paper considers a spherical underwater robot as our platform [34,35,36,37]. A spherical robot may move slower than a torpedo-shaped underwater vehicle. However, due to the high water pressure resistance of spherical objects, a spherical robot can perform rotational motions with a 0 degree turn radius.

Let *T* denote a sampling interval in discrete time systems. Let *r* denote the robot moving in 2D environments. We assume that the robot can localize itself and can access the goal’s location. For instance, Visual-Inertial Simultaneous Localization And Mapping (VI-SLAM) [38,39] or monocular SLAM [40,41,42] can be applied for robot localization in real-time.

Let $g\in R^{2}$ denote the 2D position of the goal in the inertial reference frame. Let $r\left(k\right)\in R^{2}$ denote the 2D position of the robot at time step *k* in the inertial reference frame. Let $v\left(k\right)\in R^{2}$ denote the velocity vector of the robot at time step *k*. The motion dynamics of the robot *r* are given as

(1) $$\begin{array}{c} r(k+1)=r(k)+v(k)\times T. \end{array}$$

By changing the robot’s thruster’s rotation direction, the robot can change its velocity vector v(k) at time step *k*. The motion model in (1) has been widely applied in the literature on robot controls [43,44,45,46,47,48,49,50].

The references [34,35,36] showed that by adopting vectored water-jets, a spherical underwater robot can maneuver freely in any direction. Since a spherical robot is highly maneuverable, the simple process model in (1) is feasible.

In order to move towards the goal, the robot sets its velocity vector as

(2) $$\begin{array}{c} v\left(k\right)=\frac{g-r(k)}{∥g-r(k)∥}\times S, \end{array}$$

where *S* denotes the speed of the robot.

While the robot moves towards its goal, it may detect a net using its camera. We can apply deep neural networks, such as YOLOv5 [19], for underwater net detection. Note that underwater net detection under camera measurements is not our novel contribution.

For enhancing the underwater net detection ability, we can apply various image enhancement operations [51], as well as image data augmentation techniques [52]. However, underwater net detection using camera sensors is not trivial in practice. Thus, whenever a net is detected using the robot’s camera, the robot moves away from the detected net abruptly. For moving away from the detected net, the robot uses the bearing angle of the net with respect to the robot’s camera.

Recall that Figure 2 depicts the bearing angle of the net with respect to the robot’s camera. In this figure, *b* denotes the bearing angle of the net with respect to the robot’s camera. A bold plaid indicates the net in the camera image. A bounding box containing the detected net is plotted with a dotted rectangle. In order to move away from the detected net, the spherical robot at r(k) moves in the dashed arrow direction.

After the robot moves backward for a certain distance, the robot makes a large circular turn to approach the goal, while avoiding the net. A large circular turn is desirable, since moving close to a net is dangerous for the robot.

Algorithm 1 presents the proposed net avoidance controls. In this algorithm, $RotAngle\in \{\frac{\pi }{2},-\frac{\pi }{2}\}$ indicates the rotation direction of the robot, while circling around a detected net. Both directions of movement can avoid obstacles. However, considering a net which is partially observable by the robot, the robot cannot access which maneuver leads to net avoidance. In Algorithm 1, we set $RotAngle=\frac{\pi }{2}$ initially.

In Algorithm 1, the robot stores the recent trajectory of the robot. Let $k_{d}>0$ denote a tuning parameter determining the storage window size. At each time step *k*, the stored trajectory list is given as

(3) $$\begin{array}{c} \left[r\left(k-k_{d}\right),r\left(k-k_{d}+1\right),\ldots ,r\left(k\right)\right] \end{array}$$

Here, $r\left(k-k_{d}\right)\in R^{2}$ is called the *reset point*.

We say that the robot is *stuck*, in the case where every robot position in

(4) $$\begin{array}{c} \left[r\left(k-\frac{k_{d}}{2}\right),r\left(k-\frac{k_{d}}{2}+1\right),\ldots ,r\left(k\right)\right] \end{array}$$

is inside a circle with radius β≥0. Here, β is a tuning parameter determining the stuck situation. As β decreases, the robot is stuck in a smaller space.

Once the robot is stuck, it moves towards the reset point until reaching the reset point. As the robot reaches the reset point, it moves towards the goal. Suppose that the robot has been using RotAngle as its maneuver strategy before it enters the stuck situation. In order to find a way to get out of the stuck situation, the robot changes its maneuver strategy using (RotAngle=−RotAngle). In this way, as the robot detects a net during its maneuver, it circles around the detected net in the reverse direction. The effect of this reverse maneuver strategy is presented in Section 3.2.3.

In Algorithm 1, the robot moves towards the goal by setting its velocity vector as (2). In Algorithms 1 and 2, HalfCircleMove(RotAngle) is performed whenever the robot detects a net during its maneuver.   
**Algorithm 1** Net avoidance controls  1:The robot moves towards the goal;  2:$RotAngle=\frac{\pi }{2}$;  3:**repeat**  4:   **if** the robot detects a net during its maneuver **then**  5:     HalfCircleMove(RotAngle);  6:     The robot moves towards the goal;  7:   **end if**  8:   **if** the robot is stuck **then**  9:     The robot moves towards the reset point until reaching the reset point;10:     Once the robot reaches the reset point, it moves towards the goal;11:     RotAngle=−RotAngle;12:   **end if**13:**until** The robot reaches its goal;

**Algorithm 2** HalfCircleMove(RotAngle)
1:The robot moves away from a detected net for *D* distance units;2:The robot moves along a half circle with radius *D*;3:**if** the robot detects a net during its maneuver **then**4:   HalfCircleMove(RotAngle);5:
**end if**



In Algorithm 2, the robot moves away from a detected net for *D* distance units. Here, *D* is a tuning constant, presenting the maximum sensing range of the robot. In simulations, we check the effect of changing *D*.

Let $N\in R^{2}$ denote the 2D position of the net detected by the robot. Note that the robot cannot access N using its camera measurements. The robot can only access the unit vector $\frac{r(k)-N}{∥r(k)-N∥}$ using the net’s bounding box in the camera image. In Figure 2, a dashed arrow indicates the direction associated to $\frac{r(k)-N}{∥r(k)-N∥}$. Note that the relative distance ∥N−r(k)∥ cannot be provided using passive camera sensors.

By reversing the robot’s thruster’s rotation direction, the robot can move backwards. In order to move away from N, the robot sets its velocity vector as

(5) $$\begin{array}{c} v\left(k\right)=\frac{r(k)-N}{∥r(k)-N∥}\times S. \end{array}$$

The robot moves away from a detected net for *D* distance units, by setting its velocity vector as (5) for $\frac{D}{S}$ s.

In Algorithm 2, the robot moves along a half circle with radius *D*. Before addressing the robot’s velocity vector for this circling maneuver, we define the rotation matrix M(RotAngle) as

(6) $$\begin{array}{c} M\left(RotAngle\right)=\left(\begin{array}{c} \cos(RotAngle),\sin(RotAngle)\\ -\sin(RotAngle),\cos(RotAngle) \end{array}\right). \end{array}$$

Here, M(RotAngle) indicates the rotation matrix of RotAngle in radians. Let $Q\in R^{2}$ denote the robot’s 2D location at the moment when the robot begins moving away from a detected net for *D* distance units. The robot can access Q, since the robot can localize itself using various localization methods, such as VI-SLAM [38,39] or monocular SLAM [40,41,42]. By setting the robot’s velocity vector as

(7) $$\begin{array}{c} v\left(k\right)=S\times M\left(RotAngle\right)\times \frac{Q-r(k)}{∥Q-r(k)∥}, \end{array}$$

the robot moves along a circle with radius *D*. The robot moves along a half circle with radius *D*, by setting its velocity vector as (7) for $\frac{\pi \times D}{S}$ s.

In (7), $RotAngle\in \{\frac{\pi }{2},-\frac{\pi }{2}\}$ indicates the rotation direction of the robot, while moving along a half circle with radius *D*. Here, $RotAngle=\frac{\pi }{2}$ implies that the robot moves along a half circle with radius *D* in the counter-clockwise direction. In addition, $RotAngle=-\frac{\pi }{2}$ implies that the robot moves along a half circle with radius *D* in the clockwise direction. In Algorithm 2, RotAngle is reversed only in the case where the robot is stuck. Here, RotAngle is reversed to make the robot remove itself from the stuck situation.

**Figure 3:**
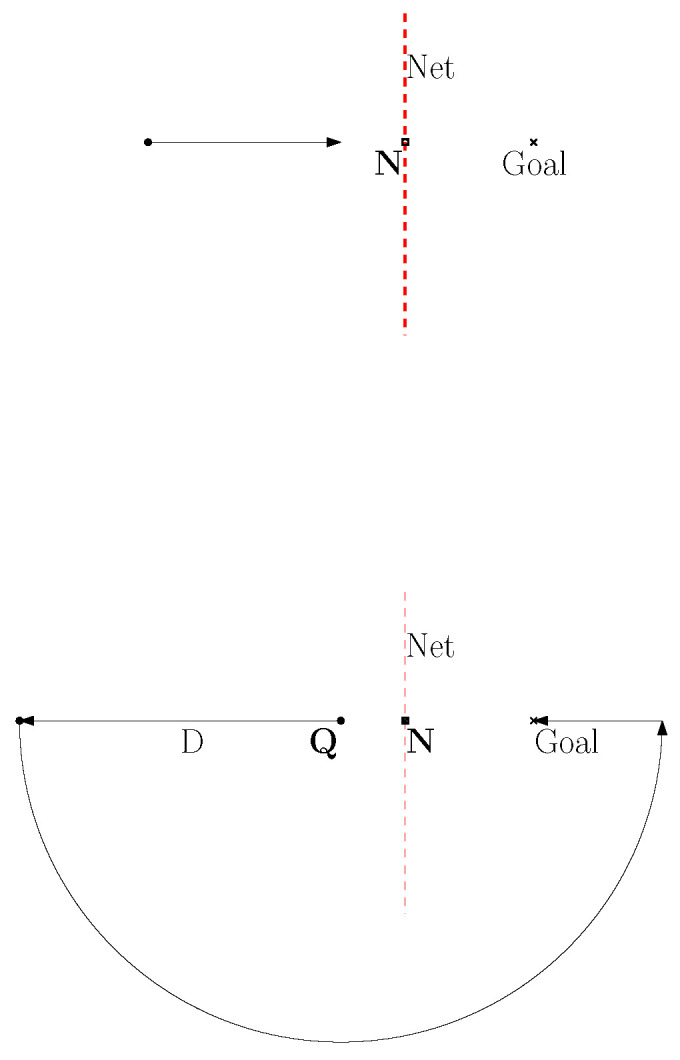
Example of net avoidance controls. A red dashed line segment indicates a net. The goal is marked with a cross. The top subplot shows the case where the robot detects the net, while moving towards the goal. The below subplot shows the case where the robot moves away from the detected net for *D* distance units, followed by moving along a half circle with radius *D*. Then, the robot can move towards the goal without being entangled by nets.

Figure 3 shows an example of net avoidance controls. A red dashed line segment indicates a net. The goal is marked with a cross. The top subplot shows the case where the robot detects the net, while moving towards the goal. The below subplot shows the case where the robot moves away from the detected net for *D* distance units, followed by moving along a half circle with radius *D*. Then, the robot can move towards the goal without being entangled by nets.

### Discussion

We acknowledge that the proposed net avoidance controls are not optimal, since the robot does not move along a shortest path to the goal. However, we argue that finding an optimal path is not possible, since the robot moves in unknown underwater environments. An optimal path can be generated only when we have a priori knowledge on obstacle environments.

Moreover, the robot cannot detect the accurate position of underwater nets using camera sensors with limited field of view. The robot can only measure the bearing angle of the detected net, with respect to its camera. Note that our collision avoidance control laws are based on the net’s bearing direction, which is computed based on the net’s bounding box. As far as we know, our paper is unique in addressing how to reach the goal, while avoiding collision with nets detected using passive cameras.

## 3. MATLAB Simulations

### 3.1. Net Detection Experiments

We show that deep neural networks can be trained to detect a fishing net. We applied YOLOv5 [19] for underwater net detection. Note that underwater net detection under camera measurements is not our novel contribution. For net detection, we can apply other types of neural networks, such as Fast R-CNN [15], Faster R-CNN [16], or Mask R-CNN [17].

In order to generate datasets related to underwater nets, we do experiments in the stream. In YOLOv5 [19], the neural network model is trained using 700 fishing net images with a validation set of 1000 images. The models are evaluated on a testing set of size 1000 images. Each image size is 640×640. The neural network is optimized using stochastic gradient descent, which has a learning rate of 0.01. Another parameter is weight decay, which is set to 0.0005 with 93.7% momentum. The batch size and epochs are 256 and 600, respectively. We used GPU (Nvidia Tesla V100 32GB) for our experiments.

Using YOLOv5 [19], we reached 0.95 mean Average Precision (mAP), when the threshold of IOU is set to 0.5. This implies that the proposed net detection can be applied in practice. We achieved 33 frames per second (FPS); thus, real-time net detection is feasible. Figure 1 shows the detection results of YOLOv5, which is applied to experiments in the stream.

### 3.2. Net Avoidance Simulations

Using MATLAB simulations, we verify the effectiveness of the proposed net avoidance controls (Algorithm 1). To the best of our knowledge, collision avoidance methods in the literature assumed that the distance between the robot and an obstacle can be accurately measured. This implies that the collision avoidance controls in the literature require that the robot has range sensors. However, in our paper, the robot only detects the bearing angle of a net, with respect to the robot’s passive camera.

The goal is located at the origin. The motion dynamics of the robot are given in (1), and the robot’s speed is S=0.5 m/s. Recall we consider a spherical underwater robot as our platform [34,35,36,37]. Thanks to the high water pressure resistance of spherical objects, a spherical robot can perform rotational motions with a 0 degree turn radius. By adopting vectored water-jets, a spherical underwater robot can maneuver freely in any direction [34,35,36].

We assume that the robot is entangled by a net, in the case where the distance from the net and the robot is less than 0.1 m. In (3) and (4), we use $k_{d}=400$ time steps and β=20 (m). These parameters ($k_{d}=400$ and β=20) are used in all simulations.

In practice, the robot may not detect a net, even in the case where the net is within the camera sensing range. Light, viewpoint, and sea floor condition can decrease the net detection probability in practice. We assume that the robot detects a net with probability $p_{d}<1$, as long as the relative distance between the robot and a point in the net is less than *D*. This implies that a net is found with probability $p_{d}<1$, as long as the net is within *D* distance from the robot. We use D=15 (m) and $p_{d}=0.9$.

We ran 30 Monte-Carlo (MC) simulations to verify the outperformance of Algorithm 1 rigorously. In every MC simulation, we randomly set the initial location of the robot in the box with size 1000×700 in meters. Per each MC simulation, the robot approached the goal, while avoiding collision with detected nets. We randomly changed the initial location, since this random initialization provides robust performance analysis of the proposed algorithm. If we fix the initial robot location in all MC simulations, then the trajectory of the robot does not change at every MC simulation.

As specific quantitative metrics to evaluate the effectiveness of the net avoidance control strategy, we use the average travel distance in every MC simulation and the computation time for all MC simulations. For convenience, avgDist denotes the average travel distance of a robot in every MC simulation. Let compTime denote the computation time of all MC simulations. It is desirable that both avgDist and compTime are as short as possible.

**Figure 4:**
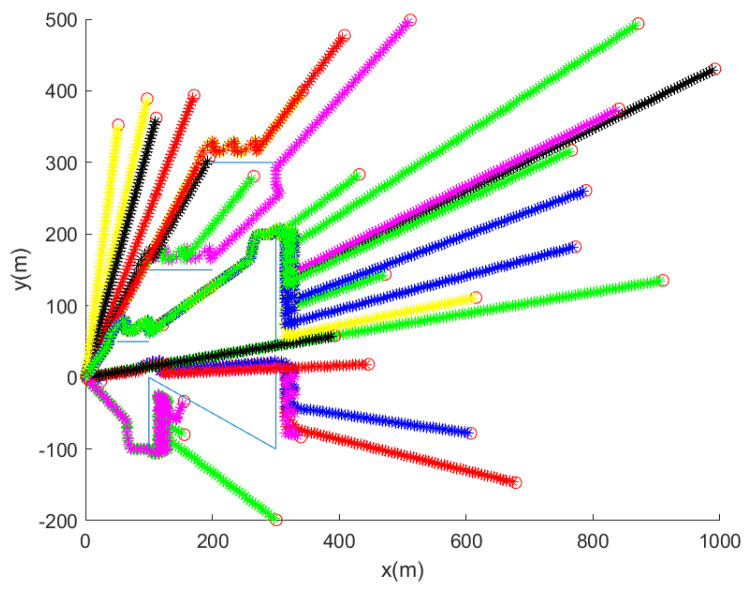
We set D=15 (m) and $p_{d}=0.9$. This plot shows the trajectory of the robot for 30 MC simulations. Per each MC simulation, the initial position of the robot is marked with a red circle. In each MC simulation, the trajectory of the robot at every 10 s is marked with asterisks of distinct colors. Blue line segments indicate underwater nets. See that the robot reaches the goal, while avoiding collision with underwater nets.

Considering the case where D=15 (m) and $p_{d}=0.9$, Figure 4 shows the trajectory of the robot for 30 MC simulations. Whenever the robot is entangled by a net, the associated MC simulation ends. In Figure 4, the initial position of the robot is marked with a red circle. In each MC simulation, the trajectory of the robot at every 10 s is marked with asterisks of distinct colors. Blue line segments indicate underwater nets. Figure 4 shows that the robot reaches the goal in all MC simulations, while avoiding collision with nets.

From Figure 4 where D=15 (m) and $p_{d}=0.9$, we obtain avgDist as 699 m. compTime is 16 s. The proposed net avoidance controls are simple, and are suitable for real-time embedded system applications.

#### 3.2.1. The Effect of Changing the Maximum Sensing Range *D*

In clear water, the maximum sensing range *D* can be large. But, in dark and dirty water, *D* can be small. We further present the effect of changing *D*. We set D=5 (m) and $p_{d}=0.9$.

**Figure 5:**
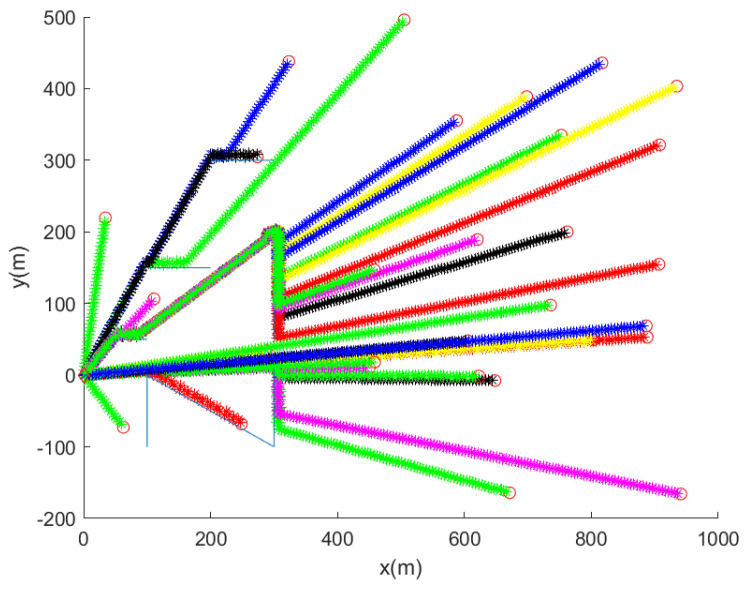
We set D=5 (m) and $p_{d}=0.9$. This plot shows the trajectory of the robot for 30 MC simulations. Per each MC simulation, the initial position of the robot is marked with a red circle. In each MC simulation, the trajectory of the robot at every 10 s is marked with asterisks of distinct colors. Blue line segments indicate underwater nets. See that the robot reaches the goal, while avoiding collision with underwater nets.

Considering the case where D=5 (m) and $p_{d}=0.9$, Figure 5 shows the trajectory of the robot for 30 MC simulations. The initial position of the robot is marked with a red circle. In each MC simulation, the trajectory of the robot at every 10 s is marked with asterisks of distinct colors. Blue line segments indicate underwater nets. Figure 5 shows that the robot reaches the goal, while avoiding collision with nets.

From Figure 5 where D=5 (m) and $p_{d}=0.9$, we obtain avgDist as 746 m, and compTime is 23 s. Recall that as we use D=15 (m) and $p_{d}=0.9$, we obtain avgDist as 699 m (Figure 4), and compTime is 16 s. See that as *D* decreases to 5 (m), both avgDist and compTime increase. This implies that the robot can reach its goal fast, as the robot is in clear water, compared to the case where the robot is in dark and dirty water.

#### 3.2.2. The Effect of Changing the Detection Probability $p_{d}$

We next check the effect of changing the detection probability $p_{d}$. In dark, dirty, and cluttered underwater environments, $p_{d}$ can be small. We set $p_{d}=0.7$, while setting D=15 m.

**Figure 6:**
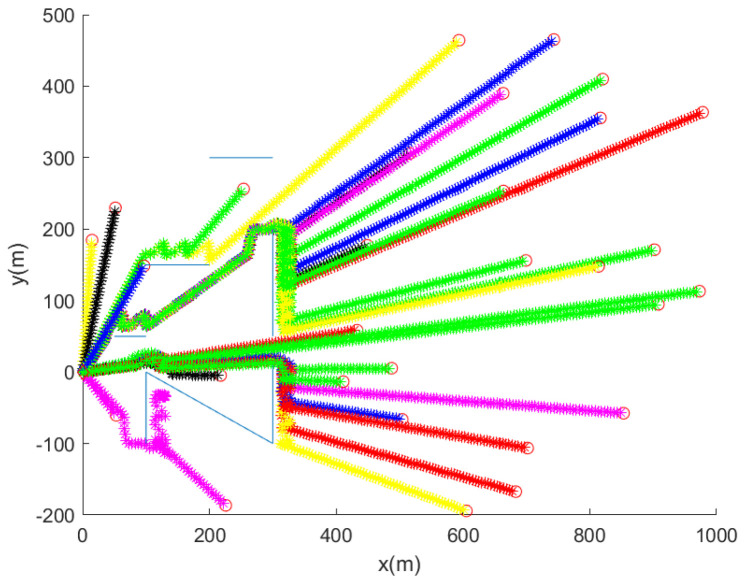
We set $p_{d}=0.7$, while setting D=15 m. This plot shows the trajectory of the robot for 30 MC simulations. Per each MC simulation, the initial position of the robot is marked with a red circle. In each MC simulation, the trajectory of the robot at every 10 s is marked with asterisks of distinct colors. Blue line segments indicate underwater nets. See that the robot reaches the goal, while avoiding collision with underwater nets.

As we set D=15 (m) and $p_{d}=0.7$, Figure 6 shows the trajectory of the robot for 30 MC simulations. The initial location of the robot is marked with a red circle. In each MC simulation, the trajectory of the robot at every 10 s is marked with asterisks of distinct colors. Blue line segments indicate underwater nets. Despite the setting of low $p_{d}$, the robot reaches the goal in all MC simulations, while avoiding collision with nets.

From Figure 6 where D=15 (m) and $p_{d}=0.7$, we obtain avgDist as 746 m, and compTime is 32 s. Recall that as we use D=15 (m) and $p_{d}=0.9$, we obtain avgDist as 699 m (Figure 4), and compTime is 16 s. See that as $p_{d}$ decreases to 0.7, both avgDist and compTime increase. This implies that the robot can reach its goal fast, as $p_{d}$ is large, compared to the case where $p_{d}$ is small.

#### 3.2.3. The Effect of Using the Strategy for Getting Out of the Stuck Situation

Once the robot is stuck, it moves towards the reset point until reaching the reset point. Then, the robot changes its maneuver strategy (RotAngle=−RotAngle) for getting out of the stuck situation. We next verify the effect of using this strategy for getting out of the stuck situation.

Suppose that one does not apply the strategy for getting out of the stuck situation. We do not apply this strategy by setting β=0. Recall that the robot is stuck, in the case where every robot position in (4) is inside a circle with radius β≥0.

**Figure 7:**
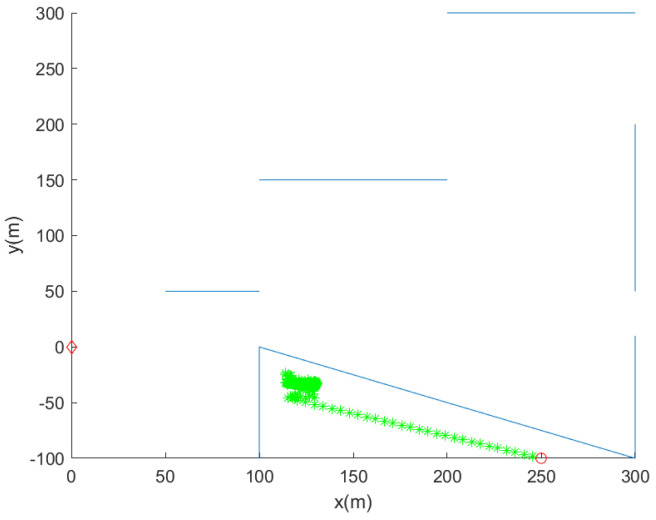
One does not apply the strategy for getting out of the stuck situation. We do not apply this strategy by setting β=0. We further set $p_{d}=0.9$, while setting D=15 m. The initial position of the robot is marked with a red circle. The trajectory of the robot at every 10 s is marked with asterisks of distinct colors. Blue line segments indicate underwater nets. See that the robot cannot get out of the stuck situation.

Figure 7 shows the trajectory of the robot, as we do not apply the strategy for getting out of the stuck situation. See that the robot cannot get out of the stuck situation. In Figure 7, we set $p_{d}=0.9$, while setting D=15 m.

**Figure 8:**
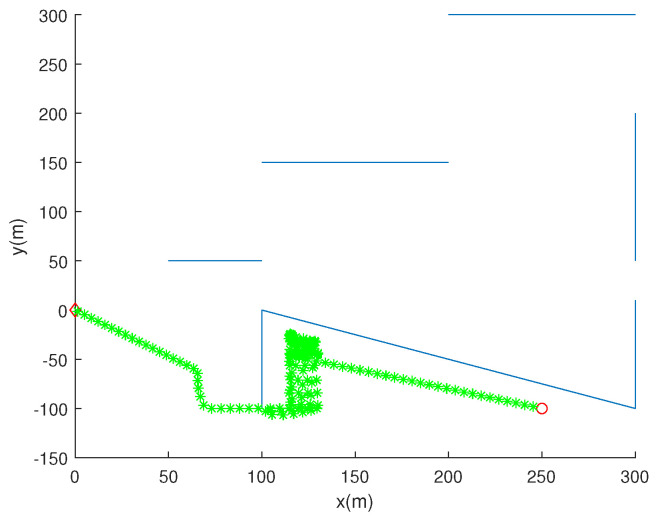
One applies the strategy for getting out of the stuck situation. We apply this strategy by setting β=20 (m). We also set $p_{d}=0.9$, while setting D=15 m. The initial position of the robot is marked with a red circle. The trajectory of the robot at every 10 s is marked with asterisks of distinct colors. Blue line segments indicate underwater nets. See that the robot reaches the goal after getting out of the stuck situation.

Considering the scenario in Figure 7 (D=15 (m) and $p_{d}=0.9$), Figure 8 shows the trajectory of the robot, as we apply the strategy for getting out of the stuck situation. We apply this strategy by setting β=20 (m). See that the robot reaches the goal after getting out of the stuck situation.

## 4. Conclusions

This paper proposes camera-based fishing net avoidance controls. Passive camera sensors do not provide the distance information between the robot and a net. Camera sensors generate the bearing angle of a net, with respect to the robot’s camera pose. Whenever a net is detected by the robot’s camera, the robot avoids the detected net by moving away from the net. Here, the bounding box of the net image is used to make the robot move away from the net.

After the robot moves backwards for a while, it makes a large circular turn followed by heading towards its goal. A large circular turn is applied, since moving close to a net is dangerous for the robot. To the best of our knowledge, our article is novel in addressing reactive control laws for approaching the goal, while avoiding nets detected using cameras.

In practice, we may have a partial information on the underwater workspace. For instance, we may have a priori information on underwater terrain environments. Based on partially known underwater environments, we can generate a shortest path from the start to the goal using various path planners, such as A-star or Dijkstra algorithms [53]. We then set waypoints along the shortest path. In order to make the robot move from one waypoint to the next one, we can use the proposed control laws. In other words, our control laws can be used to move from one waypoint to the next one, while avoiding collision with underwater nets.

The effectiveness of the proposed net avoidance controls is verified using experiments and simulations. The proposed net avoidance controls are simple, and are suitable for real-time embedded system applications. In the future, we will verify the proposed net avoidance controls by doing experiments with real underwater robots. Also, in the future, we will extend the proposed controls to multi-robot systems [54,55,56], so that a group of multiple underwater robots can move towards a goal while avoiding underwater nets.

## Data Availability

Data are contained within the article.
